# Diethyl [2,2,2-trifluoro-1-phenyl­sulfonyl­amino-1-(trifluoro­meth­yl)eth­yl]phospho­nate

**DOI:** 10.1107/S1600536808020175

**Published:** 2008-07-05

**Authors:** Sanjeeva J. Wijeyesakere, Faik A. Nasser, Jeff W. Kampf, Alexey Y. Aksinenko, Vladimir B. Sokolov, Vladimir V. Malygin, Galina F. Makhaeva, Rudy J. Richardson

**Affiliations:** a University of Michigan, Toxicology Program, 1420 Washington Heights, Ann Arbor, MI 48109-2029, USA; b University of Michigan, Department of Chemistry, 930 N. University, Ann Arbor, MI 48109-1055, USA; c Institute of Physiologically Active Compounds, Russian Academy of Sciences, Chernogolovka, Moscow Region 142432, Russian Federation

## Abstract

The title compound, C_13_H_16_F_6_NO_5_PS, is of inter­est with respect to inhibition of serine hydro­lases. Its structure contains a 1.8797 (13) Å P—C bond and two inter­molecular N—H⋯O=P hydrogen bonds, resulting in centrosymmetric dimers. An intra­molecular N—H⋯O=P hydrogen bond is also present.

## Related literature

For related literature, see: Chekhlov *et al.* (1995 ▶); Makhaeva *et al.* (2005 ▶); Adams *et al.* (2008 ▶); Chen *et al.* (2008 ▶); Guo *et al.* (2008 ▶); Kachkovskyi & Kolodiazhnyi (2007 ▶); Liu *et al.* (1995 ▶).
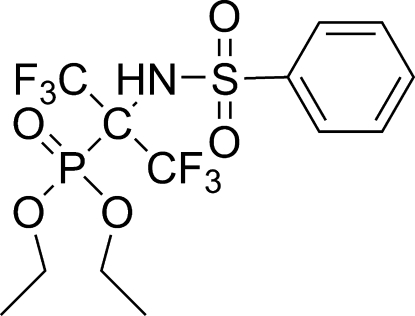


## Experimental

### 

#### Crystal data

C_13_H_16_F_6_NO_5_PS
*M* *_r_* = 443.3
Monoclinic, 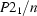

*a* = 11.6913 (15) Å
*b* = 10.1375 (13) Å
*c* = 15.5955 (19) Å
β = 93.264 (2)°
*V* = 1845.4 (4) Å^3^
*Z* = 4
Mo *K*α radiation
μ = 0.34 mm^−1^
*T* = 113 (2) K
0.60 × 0.42 × 0.40 mm

#### Data collection

Bruker SMART CCD area-detector diffractometer
Absorption correction: multi-scan (*SADABS*; Sheldrick, 2003 ▶) *T*_min_ = 0.820, *T*_max_ = 0.874
20001 measured reflections
4568 independent reflections
4027 reflections with *I* > 2σ(*I*)
*R*_int_ = 0.022

#### Refinement

*R*[*F* ^2^ > 2σ(*F* ^2^)] = 0.029
*wR*(*F* ^2^) = 0.082
*S* = 1.03
4568 reflections
246 parameters
H-atom parameters constrained
Δρ_max_ = 0.34 e Å^−3^
Δρ_min_ = −0.33 e Å^−3^

### 

Data collection: *SMART* (Bruker, 1998 ▶); cell refinement: *SAINT* (Bruker, 2003 ▶); data reduction: *SAINT*; program(s) used to solve structure: *SHELXTL* (Sheldrick, 2008 ▶); program(s) used to refine structure: *SHELXTL*; molecular graphics: *SHELXTL*; software used to prepare material for publication: *SHELXTL*.

## Supplementary Material

Crystal structure: contains datablocks global, I. DOI: 10.1107/S1600536808020175/si2094sup1.cif

Structure factors: contains datablocks I. DOI: 10.1107/S1600536808020175/si2094Isup2.hkl

Additional supplementary materials:  crystallographic information; 3D view; checkCIF report

## Figures and Tables

**Table 1:** Hydrogen-bond geometry (Å, °)

*D*—H⋯*A*	*D*—H	H⋯*A*	*D*⋯*A*	*D*—H⋯*A*
N1—H1*A*⋯O3	0.88	2.34	2.8730 (14)	119
N1—H1*A*⋯O3^i^	0.88	2.00	2.8324 (14)	158

Symmetry code: (i) 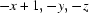.

**Fig. 1.**
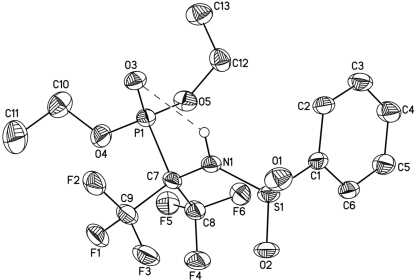
Structure of diethyl-FAP showing the atom numbering scheme. The intramolecular hydrogen bond is shown as a dashed line. Ellipsoids represent 50% occupancy.

**Fig. 2.**
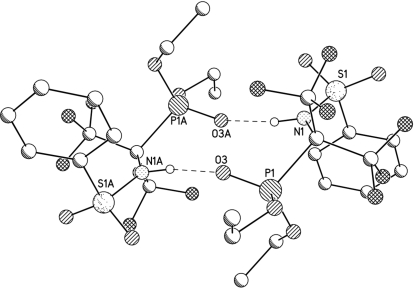
The dimer of diethyl-FAP, showing the intermolecular hydrogen bonds and the atom labelling scheme.

## supplementary crystallographic information

### Comment

The title compound is a member of the fluorinated α-aminophosphonate (FAP) group of compounds [(RO)_2_P(O)C(CF_3_)_2_NHS(O)_2_C_6_H_5_; *R* = CH_3_, C_2_H_5_, C_3_H_7_, *iso*-C_3_H_7_, *n*-C_4_H_9_, *iso*-C_4_H_9_, *iso*-C_5_H_11_, *n*-C_5_H_11_, and *n*-C_6_H_13_] that have been synthesized and used in biochemical studies as inhibitors of serine hydrolases (Chekhlov *et al.*, 1995; Makhaeva *et al.*, 2005). These studies suggested the hypothesis that inhibition of serine hydrolases by FAP compounds occurs *via* scission of the P—C bond to organophosphorylate the active site serine (Makhaeva *et al.*, 2005). Although P—C bonds are exceptionally stable in most phosphonates, enzymes such as bacterial carbon-phosphorus lyase are capable of catalyzing their cleavage, thus providing a potential method for destroying toxic phosphonates that might otherwise accumulate in the environment (Adams *et al.*, 2008). Moreover, the structure of diisopentyl-FAP revealed a 1.888 (4) Å P—C bond (Chekhlov *et al.*, 1995), which was calculated to be longer and weaker than P—C bonds in phosphonates lacking adjacent –CF_3_ groups (Makhaeva *et al.*, 2005).

To provide a further test of our hypothesis, the *X*-ray crystal structure of the title compound was determined (Fig 1). The title compound contains an intramolecular P=O···H—N hydrogen bond (Fig. 1; Table 1), and in the crystal it is linked *via* two intermolecular P=O···H—N hydrogen bonds to form inversion-related dimers (Fig. 2; Table 1). As predicted, the structure of diethyl-FAP revealed an elongated P—C bond that was 1.8797 (13) Å in length, which is not significantly different from the 1.888 (4) Å P—C bond in diisopentyl-FAP (Chekhlov *et al.*, 1995). This is long compared to P—C bond lengths of 1.822 (2) Å (Chen *et al.*, 2008), 1.803 (4) Å (Guo *et al.*, 2008), 1.818 (5) Å (Kachkovskyi and Kolodiazhnyi, 2007), and 1.805 (6) Å (Liu *et al.*, 1995) reported for the crystal structures of a variety of dialkyl phosphonates lacking α-CF_3_ groups. The long P—C bond in diethyl-FAP is expected to be labile and would explain the ability of the compound to organophosphorylate and inhibit serine hydrolases as well as their ability to undergo hydrolysis to yield phosphoric acid diethyl ester and the amide, (CF_3_)_2_CH–NH–SO_2_–C_6_H_5 _(Makhaeva *et al.*, 2005).

### Experimental

The title compound was synthesized by mixing ether solutions of equimolar amounts of diethylphosphite and the sulfonylimine of hexafluoroacetone followed by subsequent recrystallization from petroleum ether.

Colorless plates of the ethyl analog were grown *via* evaporation from methanol at 22 °C. A crystal with dimensions of 0.60 × 0.42 × 0.40 mm was cut from a larger crystal and mounted on a standard Bruker *SMART* CCD-based X-ray diffractometer equipped with a LT-2 low temperature device and normal focus Mo-target X-ray tube (λ = 0.71073 Å) operated at 2000 W power (50 kV, 40 mA). X-ray intensities were measured at 113 (2) K with the detector placed 4.980 cm from the crystal. A total of 3030 frames were collected with a scan width of 0.3° in ω and φ and an exposure time of 20 sec/frame.

Data integration yielded a total of 20001 reflections to a maximum 2θ value of 56.58° of which 4568 were independent and 4343 were greater than 2 σ(I). The final cell constants were based on the xyz centroids of 6691 reflections above 10 σ(I).

### Refinement

The hydrogen atoms were treated as riding, with N—H distance = 0.88 Å and C—H  distances in the range  0.95–0.99 Å with *U*_iso_(H) = 1.2*U*_eq_(N,C), 1.5*U*_eq_(C_methyl_).

### Crystal data

**Table:**

C_13_H_16_F_6_NO_5_PS	*F*_000_ = 904
*M**_r_* = 443.3	*D*_x_ = 1.596 Mg m^−^^3^
Monoclinic, *P*2_1_/*n*	Mo *K*α radiation λ = 0.71073 Å
Hall symbol: -P 2yn	Cell parameters from 6567 reflections
*a* = 11.6913 (15) Å	θ = 2.9–28.3º
*b* = 10.1375 (13) Å	µ = 0.35 mm^−^^1^
*c* = 15.5955 (19) Å	*T* = 113 (2) K
β = 93.264 (2)º	Plate, colourless
*V* = 1845.4 (4) Å^3^	0.60 × 0.42 × 0.40 mm
*Z* = 4	

### Data collection

**Table:**

Bruker SMART CCD area-detector diffractometer	4568 independent reflections
Radiation source: fine-focus sealed tube	4027 reflections with *I* > 2σ(*I*)
Monochromator: graphite	*R*_int_ = 0.022
*T* = 113(2) K	θ_max_ = 28.3º
φ and ω scans	θ_min_ = 2.9º
Absorption correction: multi-scan(SADABS; Sheldrick, 2003)	*h* = −15→15
*T*_min_ = 0.820, *T*_max_ = 0.874	*k* = −13→13
20001 measured reflections	*l* = −20→20

### Refinement

**Table:**

Refinement on *F*^2^	H-atom parameters constrained
Least-squares matrix: full	*w* = 1/[σ^2^(*F*_o_^2^) + (0.0427*P*)^2^ + 0.6948*P*] where *P* = (*F*_o_^2^ + 2*F*_c_^2^)/3
*R*[*F*^2^ > 2σ(*F*^2^)] = 0.029	(Δ/σ)_max_ = 0.001
*wR*(*F*^2^) = 0.082	Δρ_max_ = 0.34 e Å^−^^3^
*S* = 1.03	Δρ_min_ = −0.33 e Å^−^^3^
4568 reflections	Extinction correction: none
246 parameters	

### Special details

**Table:**

Experimental. 2103 frames × 20 sec @ 4.980 cm; 0.3 ° scans in ω & φ
Geometry. All e.s.d.'s (except the e.s.d. in the dihedral angle between two l.s. planes) are estimated using the full covariance matrix. The cell e.s.d.'s are taken into account individually in the estimation of e.s.d.'s in distances, angles and torsion angles; correlations between e.s.d.'s in cell parameters are only used when they are defined by crystal symmetry. An approximate (isotropic) treatment of cell e.s.d.'s is used for estimating e.s.d.'s involving l.s. planes.
Refinement. Refinement of *F*^2^ against ALL reflections. The weighted *R*-factor *wR* and goodness of fit *S* are based on *F*^2^, conventional *R*-factors *R* are based on *F*, with *F* set to zero for negative *F*^2^. The threshold expression of *F*^2^ > σ(*F*^2^) is used only for calculating *R*-factors(gt) *etc*. and is not relevant to the choice of reflections for refinement. *R*-factors based on *F*^2^ are statistically about twice as large as those based on *F*, and *R*- factors based on ALL data will be even larger.

### Fractional atomic coordinates and isotropic or equivalent isotropic displacement parameters (Å^2^)

**Table:**

	*x*	*y*	*z*	*U*_iso_*/*U*_eq_	
P1	0.64284 (3)	0.17485 (3)	0.04487 (2)	0.02726 (9)	
S1	0.33796 (3)	0.15018 (3)	0.174771 (18)	0.02622 (8)	
N1	0.46059 (9)	0.12554 (10)	0.13045 (6)	0.0247 (2)	
H1A	0.4569	0.0699	0.0870	0.030*	
F1	0.73735 (8)	0.14398 (10)	0.24829 (6)	0.0445 (2)	
F2	0.66649 (8)	−0.02537 (9)	0.17986 (5)	0.0429 (2)	
F3	0.57711 (8)	0.05961 (9)	0.28299 (5)	0.0412 (2)	
F4	0.56725 (9)	0.33003 (9)	0.27115 (5)	0.0434 (2)	
F5	0.67125 (8)	0.38612 (9)	0.16867 (6)	0.0415 (2)	
F6	0.48768 (7)	0.39288 (8)	0.14967 (5)	0.03547 (19)	
C1	0.26341 (11)	0.26504 (13)	0.10763 (7)	0.0255 (2)	
C2	0.24216 (12)	0.23293 (14)	0.02114 (8)	0.0307 (3)	
H2A	0.2676	0.1513	−0.0009	0.037*	
C3	0.18337 (14)	0.32241 (16)	−0.03191 (9)	0.0398 (3)	
H3A	0.1682	0.3022	−0.0909	0.048*	
C4	0.14648 (15)	0.44131 (17)	0.00055 (9)	0.0435 (4)	
H4A	0.1074	0.5028	−0.0366	0.052*	
C5	0.16614 (14)	0.47135 (16)	0.08707 (9)	0.0402 (3)	
H5A	0.1391	0.5522	0.1091	0.048*	
C6	0.22553 (12)	0.38277 (14)	0.14136 (8)	0.0313 (3)	
H6A	0.2399	0.4026	0.2005	0.038*	
C7	0.57400 (11)	0.18007 (12)	0.15084 (8)	0.0258 (2)	
C8	0.57452 (12)	0.32405 (14)	0.18606 (9)	0.0326 (3)	
C9	0.63978 (13)	0.08914 (14)	0.21698 (9)	0.0342 (3)	
C10	0.86488 (14)	0.16185 (18)	0.02046 (12)	0.0464 (4)	
H10A	0.8876	0.2351	−0.0170	0.056*	
H10B	0.8423	0.0853	−0.0161	0.056*	
C11	0.96179 (17)	0.1256 (3)	0.08278 (18)	0.0793 (7)	
H11A	0.9820	0.2017	0.1194	0.119*	
H11B	1.0283	0.0995	0.0512	0.119*	
H11C	0.9386	0.0519	0.1186	0.119*	
C12	0.50917 (13)	0.29279 (16)	−0.07438 (9)	0.0367 (3)	
H12A	0.4590	0.3713	−0.0728	0.044*	
H12B	0.4613	0.2134	−0.0674	0.044*	
C13	0.56368 (16)	0.28690 (17)	−0.15912 (10)	0.0455 (4)	
H13A	0.6155	0.3622	−0.1640	0.068*	
H13B	0.5040	0.2901	−0.2058	0.068*	
H13C	0.6071	0.2046	−0.1628	0.068*	
O1	0.27788 (9)	0.02744 (10)	0.16554 (6)	0.0339 (2)	
O2	0.36007 (9)	0.20787 (10)	0.25771 (5)	0.0346 (2)	
O3	0.61539 (8)	0.04887 (9)	0.00278 (6)	0.0311 (2)	
O4	0.77052 (9)	0.20205 (12)	0.07141 (7)	0.0435 (3)	
O5	0.59785 (9)	0.29985 (10)	−0.00394 (6)	0.0348 (2)	

### Atomic displacement parameters (Å^2^)

**Table:**

	*U*^11^	*U*^22^	*U*^33^	*U*^12^	*U*^13^	*U*^23^
P1	0.02908 (17)	0.02691 (17)	0.02539 (16)	0.00270 (12)	−0.00198 (12)	−0.00504 (12)
S1	0.03576 (17)	0.02732 (16)	0.01571 (13)	0.00577 (12)	0.00261 (11)	0.00207 (10)
N1	0.0305 (5)	0.0241 (5)	0.0192 (4)	0.0051 (4)	−0.0018 (4)	−0.0048 (4)
F1	0.0422 (5)	0.0527 (5)	0.0364 (4)	0.0091 (4)	−0.0185 (4)	−0.0058 (4)
F2	0.0575 (5)	0.0330 (4)	0.0363 (4)	0.0205 (4)	−0.0127 (4)	−0.0031 (3)
F3	0.0557 (5)	0.0430 (5)	0.0236 (4)	0.0084 (4)	−0.0093 (4)	0.0041 (3)
F4	0.0591 (6)	0.0422 (5)	0.0277 (4)	0.0078 (4)	−0.0074 (4)	−0.0163 (3)
F5	0.0415 (5)	0.0342 (5)	0.0479 (5)	−0.0042 (4)	−0.0065 (4)	−0.0146 (4)
F6	0.0423 (4)	0.0229 (4)	0.0404 (4)	0.0082 (3)	−0.0050 (3)	−0.0057 (3)
C1	0.0292 (6)	0.0292 (6)	0.0181 (5)	0.0059 (5)	0.0025 (4)	0.0009 (4)
C2	0.0388 (7)	0.0329 (7)	0.0202 (5)	0.0110 (5)	0.0005 (5)	−0.0027 (5)
C3	0.0523 (9)	0.0449 (8)	0.0215 (6)	0.0175 (7)	−0.0052 (6)	−0.0026 (5)
C4	0.0550 (9)	0.0439 (8)	0.0304 (7)	0.0243 (7)	−0.0075 (6)	0.0003 (6)
C5	0.0500 (8)	0.0379 (8)	0.0320 (7)	0.0214 (7)	−0.0022 (6)	−0.0062 (6)
C6	0.0371 (7)	0.0346 (7)	0.0222 (5)	0.0100 (5)	0.0012 (5)	−0.0042 (5)
C7	0.0319 (6)	0.0237 (6)	0.0210 (5)	0.0065 (5)	−0.0062 (4)	−0.0044 (4)
C8	0.0386 (7)	0.0287 (6)	0.0294 (6)	0.0053 (5)	−0.0067 (5)	−0.0091 (5)
C9	0.0412 (7)	0.0339 (7)	0.0261 (6)	0.0100 (6)	−0.0105 (5)	−0.0032 (5)
C10	0.0367 (8)	0.0492 (9)	0.0545 (10)	−0.0068 (7)	0.0128 (7)	−0.0050 (7)
C11	0.0352 (9)	0.110 (2)	0.0913 (17)	0.0118 (11)	−0.0041 (10)	−0.0132 (15)
C12	0.0365 (7)	0.0387 (8)	0.0345 (7)	0.0026 (6)	−0.0009 (5)	0.0107 (6)
C13	0.0660 (11)	0.0390 (8)	0.0318 (7)	0.0067 (7)	0.0048 (7)	0.0056 (6)
O1	0.0433 (5)	0.0309 (5)	0.0280 (4)	−0.0005 (4)	0.0077 (4)	0.0054 (4)
O2	0.0497 (6)	0.0391 (5)	0.0150 (4)	0.0111 (4)	0.0014 (4)	−0.0011 (4)
O3	0.0386 (5)	0.0286 (5)	0.0259 (4)	0.0044 (4)	0.0006 (4)	−0.0070 (4)
O4	0.0289 (5)	0.0543 (7)	0.0466 (6)	0.0028 (5)	−0.0028 (4)	−0.0166 (5)
O5	0.0443 (6)	0.0288 (5)	0.0308 (5)	−0.0027 (4)	−0.0012 (4)	0.0026 (4)

### Geometric parameters (Å, °)

**Table:**

P1—O3	1.4632 (10)	C4—C5	1.390 (2)
P1—O4	1.5509 (11)	C4—H4A	0.9500
P1—O5	1.5545 (10)	C5—C6	1.3923 (19)
P1—C7	1.8797 (13)	C5—H5A	0.9500
S1—O2	1.4296 (9)	C6—H6A	0.9500
S1—O1	1.4321 (11)	C7—C9	1.5539 (17)
S1—N1	1.6458 (11)	C7—C8	1.5594 (17)
S1—C1	1.7629 (12)	C10—O4	1.4540 (19)
N1—C7	1.4549 (16)	C10—C11	1.496 (3)
N1—H1A	0.8800	C10—H10A	0.9900
F1—C9	1.3361 (17)	C10—H10B	0.9900
F2—C9	1.3419 (16)	C11—H11A	0.9800
F3—C9	1.3313 (18)	C11—H11B	0.9800
F4—C8	1.3359 (16)	C11—H11C	0.9800
F5—C8	1.3354 (18)	C12—O5	1.4687 (17)
F6—C8	1.3320 (16)	C12—C13	1.501 (2)
C1—C6	1.3870 (18)	C12—H12A	0.9900
C1—C2	1.3960 (16)	C12—H12B	0.9900
C2—C3	1.3838 (18)	C13—H13A	0.9800
C2—H2A	0.9500	C13—H13B	0.9800
C3—C4	1.386 (2)	C13—H13C	0.9800
C3—H3A	0.9500		
			
O3—P1—O4	117.26 (6)	F6—C8—F5	107.48 (12)
O3—P1—O5	115.62 (6)	F6—C8—F4	108.02 (11)
O4—P1—O5	106.23 (6)	F5—C8—F4	106.43 (11)
O3—P1—C7	108.97 (6)	F6—C8—C7	110.65 (10)
O4—P1—C7	102.36 (6)	F5—C8—C7	110.84 (11)
O5—P1—C7	104.94 (6)	F4—C8—C7	113.15 (11)
O2—S1—O1	120.58 (6)	F3—C9—F1	107.88 (11)
O2—S1—N1	108.97 (6)	F3—C9—F2	106.91 (12)
O1—S1—N1	105.06 (6)	F1—C9—F2	107.63 (12)
O2—S1—C1	108.97 (6)	F3—C9—C7	111.92 (11)
O1—S1—C1	106.92 (6)	F1—C9—C7	112.07 (12)
N1—S1—C1	105.30 (5)	F2—C9—C7	110.20 (10)
C7—N1—S1	130.95 (8)	O4—C10—C11	106.50 (16)
C7—N1—H1A	114.5	O4—C10—H10A	110.4
S1—N1—H1A	114.5	C11—C10—H10A	110.4
C6—C1—C2	121.60 (11)	O4—C10—H10B	110.4
C6—C1—S1	120.05 (9)	C11—C10—H10B	110.4
C2—C1—S1	118.34 (10)	H10A—C10—H10B	108.6
C3—C2—C1	118.67 (12)	C10—C11—H11A	109.5
C3—C2—H2A	120.7	C10—C11—H11B	109.5
C1—C2—H2A	120.7	H11A—C11—H11B	109.5
C2—C3—C4	120.41 (13)	C10—C11—H11C	109.5
C2—C3—H3A	119.8	H11A—C11—H11C	109.5
C4—C3—H3A	119.8	H11B—C11—H11C	109.5
C3—C4—C5	120.51 (13)	O5—C12—C13	110.09 (13)
C3—C4—H4A	119.7	O5—C12—H12A	109.6
C5—C4—H4A	119.7	C13—C12—H12A	109.6
C4—C5—C6	119.89 (13)	O5—C12—H12B	109.6
C4—C5—H5A	120.1	C13—C12—H12B	109.6
C6—C5—H5A	120.1	H12A—C12—H12B	108.2
C1—C6—C5	118.90 (12)	C12—C13—H13A	109.5
C1—C6—H6A	120.5	C12—C13—H13B	109.5
C5—C6—H6A	120.5	H13A—C13—H13B	109.5
N1—C7—C9	109.28 (11)	C12—C13—H13C	109.5
N1—C7—C8	114.70 (10)	H13A—C13—H13C	109.5
C9—C7—C8	109.24 (10)	H13B—C13—H13C	109.5
N1—C7—P1	103.16 (8)	C10—O4—P1	123.57 (10)
C9—C7—P1	110.27 (9)	C12—O5—P1	122.10 (9)
C8—C7—P1	110.04 (9)		

### Hydrogen-bond geometry (Å, °)

**Table:**

*D*—H···*A*	*D*—H	H···*A*	*D*···*A*	*D*—H···*A*
N1—H1A···O3	0.88	2.34	2.8730 (14)	119
N1—H1A···O3^i^	0.88	2.00	2.8324 (14)	158

Symmetry codes: (i) −*x*+1, −*y*, −*z*.
